# Persistent Core Populations Shape the Microbiome Throughout the Water Column in the North Pacific Subtropical Gyre

**DOI:** 10.3389/fmicb.2019.02273

**Published:** 2019-10-01

**Authors:** Daniel R. Mende, Dominique Boeuf, Edward F. DeLong

**Affiliations:** Daniel K. Inouye Center for Microbial Oceanography, Research and Education, School of Ocean and Earth Science and Technology, University of Hawai‘i at Mānoa, Honolulu, HI, United States

**Keywords:** microbial oceanography, metagenomics, microbial evolution, temporal stability, time series, water column

## Abstract

Marine microbial communities are responsible for many important ecosystem processes in the oceans. Their variability across time and depths is well recognized, but mostly at a coarse-grained taxonomic resolution. To gain a deeper perspective on ecological patterns of bacterioplankton diversity in the North Pacific Subtropical Gyre, we characterized bacterioplankton communities throughout the water column at a fine-grained taxonomic level with a focus on temporally persistent (core) populations. Considerable intra-clade microdiversity was evident in virtually every microbial clade examined. While some of the most abundant populations comprised only a small fraction of the intra-clade microdiversity, they formed a temporally persistent core within a more diverse array of less abundant ephemeral populations. The depth-stratified population structure within many phylogenetically disparate clades suggested that ecotypic variation was the rule among most planktonic bacterial and archaeal lineages. Our results suggested that the abundant, persistent core populations comprised the bulk of the biomass within any given clade. As such, we postulate that these core populations are largely responsible for microbially driven ecosystem processes, and so represent ideal targets for elucidating key microbial processes in the open-ocean water column.

## Introduction

Microbial plankton are major facilitators of energy and matter flux in the ocean, and their diversity and vertical distributions largely reflect the depth-stratified physicochemical structure of the surrounding environment. From surface waters to the base of the photic zone steep gradients in light quality and intensity, temperature, and macro- nutrient concentrations (Somero and Hochachka, 1971; Karl and Church, 2017) are typical in open ocean habitats. At greater oceanic depths, the mesopelagic zone (200–1000 m) is physically characterized by lower light intensity and temperature, increasing hydrostatic pressure, and diminishing microbial biomass and energy resources (Karl and Church, 2017) than the photic zone. These environmental gradients influence the vertical distributions of planktonic bacterial and archaeal taxa and traits. For example, in the North Pacific Subtropical Gyre (NPSG), evidence suggests that deeper dwelling microbial communities are characterized by more variable gene content and higher trait diversity, resulting in more diverse functional attributes as compared to surface water bacterioplankton (Konstantinidis et al., 2009; Bryant et al., 2012; Mende et al., 2017a). In addition, elevated macronutrient concentrations but limited energy supplies below deep chlorophyll maximum (DCM) have been implicated as drivers for the fundamentally different genome and proteome architectures of diverse microbial clades below the DCM, compared to those found in overlying surface waters (Mende et al., 2017a).

Recent studies have enhanced our understanding of the ecology of both surface and deep water bacterial and archaeal clades, including the depth-dependent variation in genetic and physiological properties among a few clades (Field et al., 1997; DeLong et al., 2006; Giovannoni et al., 2014; Biller et al., 2015; Giovannoni, 2017; Mende et al., 2017a). For example, the variable vertical distributions of different *Prochlorococcus* subclades in the water column reflect adaptations to varying light and nutrient availability in the photic zone (Rocap et al., 2003; Biller et al., 2015; Braakman et al., 2017). Similarly, SAR11, one of the ocean’s most abundant and cosmopolitan heterotrophic bacterial clades (Morris et al., 2002; Martín-Cuadrado et al., 2007; Konstantinidis et al., 2009; Schattenhofer et al., 2009; Eloe et al., 2011; Thrash et al., 2014; Giovannoni, 2017), is comprised of several different subclades, each with a distinct vertical zonation (Field et al., 1997; Carlson et al., 2009; Giovannoni and Vergin, 2012; Grote et al., 2012; Vergin et al., 2013; Thrash et al., 2014). For many other, less abundant but potentially important clades much less is known about their distribution, ecology, physiology, and functional potential.

Genomic and metagenomic surveys of oceanic microbial communities have revealed extensive population microheterogeneity among surface dwelling bacterioplankton clades (Rusch et al., 2007; Yooseph et al., 2007; Kashtan et al., 2014). Open ocean metagenomic studies have revealed differential patterns of diversity that occur in surface water vs. deeper water bacterioplankton communities (DeLong et al., 2006; Martín-Cuadrado et al., 2007; Pham et al., 2008; Konstantinidis et al., 2009; Sunagawa et al., 2015; Bryant et al., 2016; Mende et al., 2017a). In the Mediterranean Sea, recent work has confirmed discrete stratification of different bacterioplankton subclades in the upper water column, wherein specific bacterial subclades were found to be confined to discrete horizontal layers of no more than 30 m (Haro-Moreno et al., 2018). The Tara and Malaspina oceanographic expeditions have further extended our current understanding of microbial population structure, by combining global geospatial sampling with synoptic oceanographic data (Bork et al., 2015; Duarte, 2015; Lima-Mendez et al., 2015; Sunagawa et al., 2015). In addition, time-series studies are beginning to provide further insight into planktonic microbial phylogenetic diversity, temporal and seasonal variability, evolutionary processes, and community structure (Gilbert et al., 2009; Zaikova et al., 2010; Bryant et al., 2012; Cram et al., 2015; Mende et al., 2017a; Martin-Platero et al., 2018). Both spatial and temporal sampling schemes allow for the computation of core taxa and communities. Typically, core taxa revealed in spatial sampling represent higher taxonomic levels such as genera and phyla, since different locations are often inhabited by different species or strains. In contrast, temporal sampling has the potential to reveal persistent core species and strains within specific locales. For example, persistent, individual specific populations were observed in the human gut microbiome (Schloissnig et al., 2013). In marine environments, persistent taxa (defined as 97% ID 16S OTUs) have been observed as abundant, representing 35% of all sequencing reads in surface waters in the English channel over a 6 year period (Gilbert et al., 2012). More recently, fine-grained core communities of sponges (defined as 100% ID 16S rRNA OTUs) and their exchange with the surrounding seawater were examined, showing that persistent core communities represented on average 75% of the community abundance within different sponge species. For some sponge species, a large part (up to 99%) of the core community was shared with the surrounding seawater (Turon et al., 2018).

Here, we explored fine-scale bacterioplankton diversity patterns in the NPSG at Station ALOHA using a terabase scale metagenomic dataset sampled monthly at seven different depth over the course of 1.5 years (Mende et al., 2017a). We employed a modified version of the marker gene based operational taxonomic units (mOTU) approach (Sunagawa et al., 2013, 2015; Milanese et al., 2019) based on a protein-coding universal, single-copy marker gene. In this study, we focused on the use of one universal single copy marker gene, COG0012 (a ribosome-associated GTPase, see section “Materials and Methods”). All following references to mOTUs herein refer to the use of COG0012 as the universal single copy marker gene). The resulting high-resolution (approximately species-level) taxonomic profiles enabled a reference-independent investigation of species level diversity patterns among multiple microbial clades throughout the water column, from surface waters to 1000 m depth. Focusing on core-mOTUs (defined here as those mOTUs that were present in every time-series sample taken at a given depth), we interrogated the impact of these persistent populations on microbial community structure. The high abundance of specific core-mOTUs underscores their relevance in understanding bacterioplankton ecology in the open ocean water column. Further, our data and analyses revealed shared patterns of diversity and ecotypes across many different bacterioplankton clades.

## Materials and Methods

### Data

Metagenomic sequence data, sequence assemblies, gene annotations, and corresponding physicochemical data were derived from 76 samples collected between August 2010 and December 2011 at 7 depths of 25 m to 1000 m at Station ALOHA on 12 Hawaii Ocean Time-series (HOT) cruises as previously reported (Sunagawa et al., 2015; Mende et al., 2017a; Supplementary Table S1). Sequence data and assemblies are available here: https://www.imicrobe.us/#/projects/263. In addition, we obtained gene sequences from the Tara Oceans Expedition and a custom database of select marine genomes (Sunagawa et al., 2015; Mende et al., 2017a).

### mOTUs

mOTUs are sequence clusters of universal, single-copy, protein-coding marker genes (MGs) extracted from metagenomes (Sunagawa et al., 2013, 2015; Mende et al., 2017a). We established a customized version of the mOTUs using the above-mentioned dataset in combination with genes from the Tara Oceans Expedition (Sunagawa et al., 2015) and a custom database of select marine genomes (Mende et al., 2017a). Usually, the mOTUs method employs 10 multiple marker genes, but here we used only one marker gene due to the known issues when linking mOTUs from different genes (Milanese et al., 2019). Among this set of marker genes, the COG0012 gene (a ribosome-associated GTPase) has a number of beneficial traits. In earlier studies, this COG was shown to be useful for inferring phylogenetic relationships with high fidelity, yielding results that are congruent with ribosomal RNA analyses (Harris, 2003; Bern and Goldberg, 2005; Mende et al., 2013). In addition to the phylogenetic resolution COG0012 provides, its features suggest it as a useful marker for defining relationships among closely related microbial taxa. Specifically, COG0012 is present as a single copy gene, is found universally in microbial genomes reported to date (Supplementary Table S2), and has very low rate of horizontal gene transfer (Creevey et al., 2011). In conjunction with its size (Median: 1095 nucleotides, 365 amino acids), this renders COG0012 sequences useful for both broad as well as fine scale phylogenetic analyses, as does its protein-coding nature (e.g., fine-grained phylogeny can be analyzed using nucleotide sequences, while deeper phylogenetic exploration can utilize protein sequences). It is also particularly suitable for high throughput, large scale automated alignment workflows (Mende et al., 2013; Sunagawa et al., 2013). Finally, the species-level accuracy of COG0012 mOTUs demonstrates its comparability to species clusters defined using multiple conserved proteins (40 different protein-coding genes), and provides greater taxonomic resolution and fidelity among closely related clades than the 16S rRNA gene (Mende et al., 2013; Sunagawa et al., 2013; Supplementary Table S3). As a consequence, for this study, we focus on the validation and use of COG0012 mOTUs to survey the phylogenetic diversity and spatial distributions of bacterial and archaeal communities at station ALOHA. To account for the uncertainty in defining species using any single gene for lineages with different evolutionary divergence rates, we refer to the COG0012 mOTUs here as “near-species level sequence clusters” throughout the manuscript. The fetchMGs tool (Mende et al., 2013; Sunagawa et al., 2013) was used to extract the universal, single-copy COG0012 genes from the predicted genes in the above mentioned meta-genomic datasets.

The COG0012 genes were clustered into sequence clusters using a cutoff of 94.8% nucleotide identity (Sunagawa et al., 2013). This cutoff was optimized to accurately delineate bacterial species (also Supplementary Tables S2, S3), but due to the uncertainty of the microbial species definition among diverse phylogenetic clades, and our dependence on one protein coding gene (COG0012 for most analyses), the mOTUs reported in this study are referred to as “near-species level.” The complete clustering procedure was performed as following: Nucleotide identities between all pairs of orthologous sequences were calculated from pairwise alignments using vsearch (v1.9.3) retaining only alignments of 20 or more aligned bases (Rognes et al., 2016). Average linkage hierarchical clustering was applied to the resulting distance matrix and near-species level clusters were generated employing the optimized cutoff of 94.8% nucleotide ID. Low abundance mOTUs with an average coverage of 0.5× or less were removed (see below for details on abundance estimations) as were aberrant singleton mOTUs (manually curated to avoid incorrect gene prediction) yielding 2228 mOTUs. Representatives of each mOTU were selected and phylogenetic trees were reconstructed using the ete toolkit (see below for details). The representative sequences of the mOTUs and sequence alignments used in these analyses are available as Supplementary Data S5.

### COG0012 and 16S rRNA Gene Universality and Uniqueness

We assessed the COG0012 and 16S rRNA genes using the proGenomes database (Mende et al., 2017b). The database contains 25,038 high-quality (complete and draft) genomes including gene annotations. We extracted COG0012 and 16S rRNA gene counts for all genomes and calculated how many genomes had at least one copy of each gene and how many genomes had exactly one copy per genome. Results are listed in Supplementary Table S2.

### Fidelity of the 16S rRNA and COG0012 Genes for Species Delineation

The accuracy and recall of species delineation was assessed based on sequence clustering using the COG0012 and 16S rRNA genes. For this purpose, we used a curated, type-strain based taxonomy (based on the NCBI taxonomy) as published in Mende et al. (2013). The classical species delineation cutoff for 16S rRNA of 97% nucleotide ID and an optimized cutoff of 99.1% were assessed in Mende et al. (2013). We added an assessment of COG0012 using the same procedures as described in detail in Mende et al. (2013). Results are listed in Supplementary Table S3.

### Clade Designations

**FIGURE 1:**
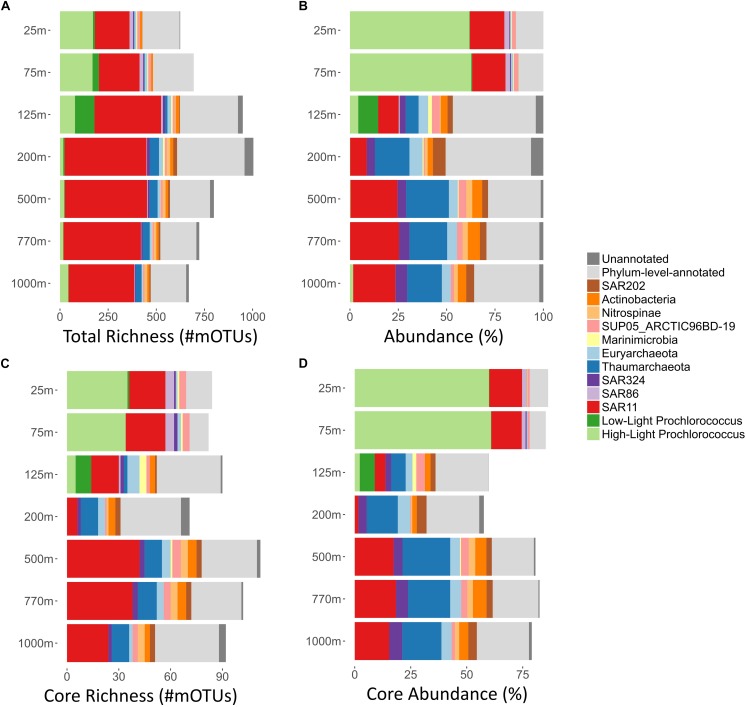
Summary of per-depth core richness, total richness, average abundances, and core abundances, of major bacterial and archaeal clades at Station ALOHA. For each depth (*y*-axis; 25–1000 m), major bacterial and archaeal clades are displayed. mOTUs that could be annotated at only the phylum level (light gray) and those that were not annotated to any known taxonomic clade (dark gray) were summarized, respectively. **(A)** Total richness of different microbial clades was calculated as described in methods and displayed as bar charts. Total richness is the number of mOTUs present in at least one sample, in any given depth. **(B)** Clade% abundances based on COG0012 mOTU abundances were first summed on a per sample basis, and then averaged across all samples of each depth. The bar charts display the total abundance in percent of the whole community for each clade at each given depth. **(C)** Core-mOTU richness of different microbial clades was calculated as described in methods and displayed as bar charts. In short, core-mOTU richness is the number of core-mOTUs that are present in every sample taken at a given depth. **(D)** Summary of per-depth average core-mOTU% abundances (of the whole community) for different bacterial and archaeal clades. For each sample core abundances were calculated as sums of the abundances (percent of the whole community) of COG0012 core-mOTUs for every bacterial and archaeal clades. Subsequently, the mean average across all samples of each depth was calculated.

Taxonomic assignments of reference genomes to major microbial clades was performed as previously described in Mende et al. (2017a). In summary, a genome-based phylogenetic tree of 389 reference genomes was reconstructed using the ete3 toolkit (Huerta-Cepas et al., 2016). Monophyletic clades of this phylogenetic tree were assigned to microbial clades *via* genomes with established clade designations and these designations were subsequently propagated to genomes within the same phylogenetic clade of the generated tree. ALOHA COG0012 mOTU genes were taxonomically annotated to one of these clade-designated genomes and other taxonomic levels (such as phyla and genera) by aligning to a custom version of RefSeq release 75 (Tatusova et al., 2015) which was amended by a number of high quality SAGs from marine environments. Alignments to the amended version of RefSeq were generated by LAST v. 756 (scoring parameters “−b 1 −x 15 −y 7 −z 25”) (Kiełbasa et al., 2011). The best scoring alignment of each gene sequence within a mOTU cluster was used to calculate a majority taxonomic assignment for each mOTU at all taxonomic levels. In addition, all marine Chloroflexi genomes were downloaded from NCBI on July 11, 2018 and manually assigned to SAR202 where applicable. ALOHA COG0012 mOTU genes were aligned to Chloroflexi and SAR202 using LAST v. 756 (scoring parameters “−b 1 −x 15 −y 7 −z 25”). These pairwise alignment-based annotations were then manually reconciled with the phylogenetic tree and displayed on the levels of phylum and “clades,” referencing to well-known marine bacterial and archaeal clades (Figure 1). Clade designations are available in Supplementary Table S4.

### ALOHA COG0012 mOTU Phylogenetic Trees

**FIGURE 2:**
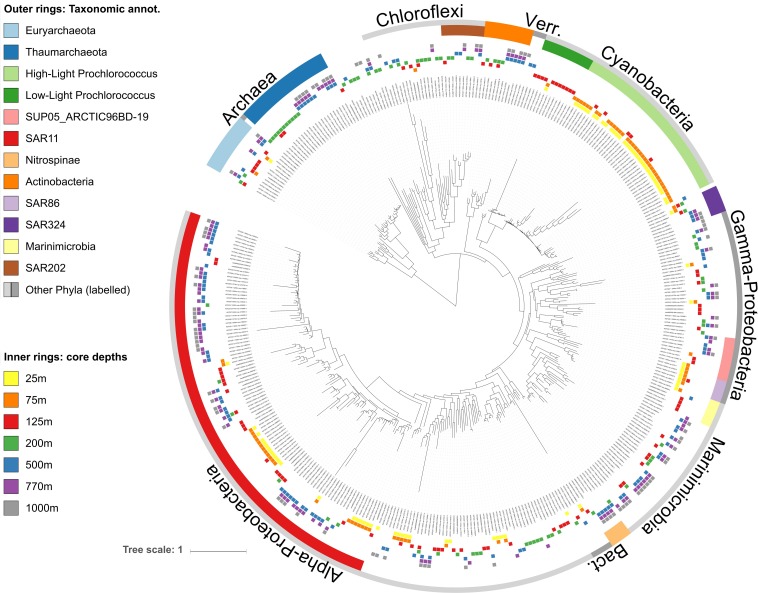
Phylogenetic tree of core-mOTUs found throughout the water column at station ALOHA. The tree was reconstructed from COG0012 sequences of 390 environmental COG0012 mOTU genes representing core-mOTUs. Bacterial and archaeal clades are labeled according to their taxonomic affiliations (outer rings, see section “Materials and Methods” for more details). Core-mOTUs are defined as those that are present in all samples (time points) of at least one depth. Inner rings show the depths in which mOTUs were persistent (present at all time points).

We constructed a phylogenetic tree of 2228 COG0012 mOTUs from Station ALOHA, along with 389 selected reference genomes (Supplementary Figure S1). More specifically, representative sequences were selected as the longest sequence of every COG0012 mOTU. COG0012 sequences from mOTUs and reference genomes were then used to construct a phylogenetic tree using ete3 (workflow “standard_trimmed_raxml_bootstrap”). The workflow consists of following steps: A multiple sequence alignment was generated using Clustal Omega (Huerta-Cepas et al., 2016; Sievers and Higgins, 2018), the alignment was trimmed using trimAl (Capella-Gutiérrez et al., 2009), optimal parameters for tree generation were calculated using PhyML 3.0 (Guindon et al., 2009), and the phylogenetic tree was generated using RAxML (Stamatakis, 2015). iTOL (Letunic and Bork, 2007, 2016) was used to display the tree. The sequences are available in Supplementary Table S4 and mappings from mOTUs to their representative sequences is available as Supplementary Table S5. The tree is available for interactive analyses at https://itol.embl.de/tree/168105215178386981532637781. The same workflow was used to generate a phylogenetic tree of all core-mOTU representatives shown in Figure 2 and this tree is available at https://itol.embl.de/tree/168105208209248051557887247.

### Mapping/Abundance Estimation

Marker gene based operational taxonomic units abundances were estimated as previously described in Mende et al. (2017a). In short, sequencing reads from station ALOHA were quality trimmed using MIRA (Chevreux, 2005) and aligned to the ALOHA mOTU sequence database using BWA (mem algorithm and standard parameters). The resulting alignments were filtered using a 95% nucleotide identity cutoff and a minimum alignment length of 45 nucleotides using msamtools (a minimum alignment length of 60 bp for partial alignments) (Arumugam et al., 2011). The best alignment for each insert was determined using BWA alignment scores. If both reads of an insert were aligned to the same reference a summed alignment score for the insert was calculated. The highest scoring alignment for each insert was kept for abundance counting and inserts with multiple highest scoring alignments were flagged as multiple mappers. To estimate the abundance of every mOTU, we first counted all unique alignments to each of the COG0012 genes of a mOTU (alignments not flagged as multiple mappers). In a second step all multiple mappers were distributed among the different mOTUs according to the mOTU abundance profiles of the unique alignments. mOTU coverage was derived by calculating the total number of bases mapping to a COG0012 gene of a mOTU and then dividing this number by the length of the respective gene. Using our approach, abundance reads are relative to total reads mapped any mOTU gene (included cultured and uncultured organisms) and these are independent of the per ml cell abundance, with that in mind for simplicity sake we will use “abundance” for “relative abundance” throughout the remainder of the text.

### mOTU Richness

COG0012 mOTU richness was calculated from read mapping count data (for this purpose only uniquely mapping inserts were used). For each sample, insert counts were down-sampled to the lowest total insert count of all samples (2747 inserts) using the function rrarefy of the R package Vegan (Dixon, 2003). In general, mOTU richness was calculated using the function specnumberof the R package Vegan. Time-series datasets allowed for the calculation of multiple cross-sample richness indices. For this purpose, read counts of each sample were down-sampled as described. From these, we calculated the “core-mOTUs” at each depth. “core-mOTUs” are defined here as those mOTUs that are present in every sample/time-point at a given depth. The core richness was then calculated as the number of core-mOTUs. The total richness for each depth was calculated in a similar manner, but as the number of mOTUs present in at least one sample of a given depth. We further calculated the richness found within clades (Figure 1) and phyla (Supplementary Figure S2) from these.

### Partial Correlation Analysis

To elucidate the relationship between core richness (including only those mOTUs that appeared in all time-series samples at any given depth), total richness and abundance, we applied classical Spearman correlation and partial correlation analysis. All analyses were performed using R (Spearman correlations: stats package; partial correlations: ppcor package) (Kim, 2015). Results are summarized in Supplementary Table S6. Although all richness estimates were based on down-sampled count data, it is possible the abundance of a phylum itself might represent a confounder for within-phylum richness estimates. This appears unlikely however, in part due to the high abundance and, in comparison, low richness of core-mOTUs (Figure 1).

## Results and Discussion

We detected a total of 2228 distinct mOTUs (Sunagawa et al., 2013, 2015; Milanese et al., 2019) over a time- and depth-series of metagenomic samples from Station ALOHA (Supplementary Table S1; Mende et al., 2017a). A phylogenetic tree of the mOTUs accurately resolved well-known and previously described bacterial and archaeal phyla and clades (Supplementary Figure S1 and Supplementary Table S4, also see section “Materials and Methods”). The vast majority of mOTUs could confidently be annotated at the phylum, genus, or clade level, revealing population level distributions within those groups (Supplementary Figure S1, also see section “Materials and Methods”).

Overall, the most numerically abundant microbial clades yielded the largest number of mOTUs (Figures 1A,B and Supplementary Figures S1, S2). The SAR11 clade was the most phylogenetically diverse clade, represented by 831 different mOTUs (38.2% of all detected mOTUs, median abundance of 17.9%). *Prochlorococcus*, found predominantly in the euphotic zone, comprised the second largest clade of mOTUs, encompassing a total of 303 mOTUs (13.6% of all detected mOTUs, median abundance: 1.7%, median epipelagic (25–125 m) abundance: 62%, median 200–500 m abundance: 0.2%). These results are consistent with earlier observations of microdiversity among *Prochlorococcus* genes and genomes, that partition them into multiple, often co-occurring population clusters (Mende et al., 2013; Kashtan et al., 2014, 2017; Biller et al., 2015).

Most other mOTUs could be assigned to known, but lower abundance clades, leaving only a small number (62 mOTUs, 2.8% of all mOTUs, median cumulative abundance: 2%) of mOTUs that could not be taxonomically assigned since they were phylogenetically distant from any known taxa currently represented in our reference databases (Figure 1B and Supplementary Figure S1).

As a whole, these results are consistent with earlier observations showing that composition of the microbial communities found at different depths at Station ALOHA is highly stratified and shows a high degree of temporal stability (DeLong et al., 2006; Bryant et al., 2016; Mende et al., 2017a). To gain additional insight into this phenomenon at a more fine-grained level, we investigated temporally persistent populations for each sampling depth. This yielded 390 core-mOTUs that were present at all time points of at least one depth (Figure 2). While these persistent mOTUs represented only 17.5% of the total mOTU richness, they were highly abundant (53–85% of the total abundance at the different sampled depths, Figures 1C,D) and represented the major clades found at Station ALOHA (Figure 2 and Supplementary Figure S1). Further, the core-mOTUs as a whole clearly captured the community structure of the total community (Supplementary Figure S3) supporting their implied importance for this ecosystem.

To study these trends in greater detail, we investigated the richness and abundance patterns of major bacterial and archaeal clades found at Station ALOHA for each sampled depth independently. To facilitate these analyses, we computed the mOTU richness of each clade at each depth across all time points in two different ways: (1) Total (cumulative) richness (the total number of all different mOTUs in a given microbial clade that occur in at least one time point at a given depth) (Figure 1A); and (2) core richness (i.e., the number of different core-mOTUs of a given depth within each clade) (Figure 1C). In addition, we calculated the average (mean) abundance for each clade at each sampled depth for the overall (total) community and the core-mOTUs (Figures 1B,D).

As anticipated from previous studies, the SAR11 clade was both highly abundant (8.6–25.3%, mean: 18%) and highly diverse at all depths (200–421 total mOTUs detected per depth, median: 350; Figures 1A,B). In addition, SAR11 was represented by core-mOTUs at every depth (6–40 core-mOTUs, median: 24), that were relatively abundant (cumulative abundance of SAR11 core-mOTUs: 1.64–18.79%, mean: 13.83% of the whole community) (Figures 1C,D, 2). Notably, SAR324 (mean abundance: 3.44% of the whole community) and Euryarchaeota (mean abundance: 3.95% of the whole community) were also represented by core-mOTUs at every depth (median: 2 and 2 core-mOTUs, respectively) (Figures 1B,C, 2). The environmental persistence and ubiquity of members of these clades throughout the water column suggests their potential ecological importance, despite their overall low abundance.

**FIGURE 3:**
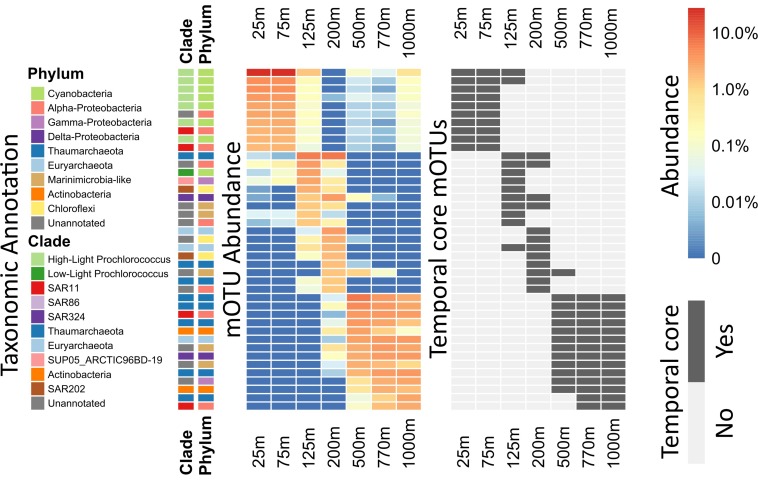
Heatmap of per-depth average abundances and core depths for highly abundant mOTUs. For each mOTU, the average across all samples of each depth was calculated. The 10 most abundant mOTUs for every depth were calculated and plotted in descending order. Side bars (on the left) show taxonomic annotations to major marine bacterial and archaeal phyla and clades. Left, the heatmap colors display the logarithm of the percent abundance of the whole community, for each clade at each given depth. Right, the heatmap indicates whether or not the displayed mOTUs are core-mOTUs for each depth. An extended abundance heatmap of this figure is available (Supplementary Figure S4).

In contrast, high-light *Prochlorococcus* mOTUs were most abundant in all samples above 125 m (mean abundance: 62.25% of the whole community) as expected, and diminished sharply in relative representation at greater depths. At 25 and 75 m, we found 33 and 35 high-light *Prochlorococcus* core-mOTUs, respectively, representing a large proportion of all core-mOTUs of all clades at these depths (79 and 82, respectively), but only a small proportion of all high-light *Prochlorococcus* mOTUs found at these depths (164 and 167 total *Prochlorococcus* mOTUs at 25 and 75 m, respectively) (Figures 1, 2). Unexpectedly however, high-light *Prochlorococcus* mOTU types were found consistently at depths of 500–1000 m throughout the time-series (Figures 1, 3 and Supplementary Figure S4), albeit at drastically lower abundances than in surface waters. This was not the case for samples from 200 m depth.

Rank abundance plots showed that 8 of the 10 most abundant *Prochlorococcus* mOTUs found at 25 m were also among the 10 most abundant *Prochlorococcus* mOTUs found at mesopelagic depths (500–1000 m) (Supplementary Figure S5). Our observations here likely reflect previously postulated positive correlation patterns that are predicted between the magnitude of picophytoplankton productivity, and the level of its export to the deep sea (Richardson and Jackson, 2007). Consistent with these results, *Prochlorococcus* was also recently observed in poisoned sediment trap samples that collected particles at 500 m in the NPSG (Fontanez et al., 2015). These results highlight the utility of mOTUs for tracking specific populations of bacterioplankton throughout the water column, and determining their autochthonous or allochthonous origins. Since the *Prochlorococcus* phylotypes found in the mesopelagic were identical to those most abundant in surface waters, it seems clear that they represent non-living cells transported from the epipelagic to the mesopelagic.

In contrast to *Prochlorococcus* which was mainly found in the sunlit zone, thaumarchaeotal mOTUs were detected consistently at high abundances in deeper waters (≥200 m), and more sporadically and at much lower abundance at 125 m (Figures 1, 3). These observations are consistent with earlier studies (e.g., Santoro et al., 2015), and with the persistently stratified water column of the NPSG. The underexplored SAR202 clade (Marine Chloroflexi) was found at similar depths as Thaumarchaeota, but was found in highest abundance at 200 m depth (Supplementary Figure S6; Morris et al., 2004; Varela et al., 2008). Though microbial communities found around 200 m depth had the lowest cumulative abundance of persistent core-mOTUs (53%, possibly implying a temporarily variable environment), our analyses did reveal a number of persistent Chloroflexi mOTUs (Figures 1–3 and Supplementary Figure S7), many of which could be reliably annotated as members of the SAR202 clade. Previous results indicate SAR202 populations to have a highly flexible genetic potential for the degradation of organic molecules which is consistent with the potential of detritus as a major energy source at 200 m (Landry et al., 2017; Mehrshad et al., 2018). Though representatives of the SAR202 clade were also detected in deeper mesopelagic waters, they were phylogenetically separate (Figure 2) indicating the existence of persistent, depth-specific SAR202 ecotypes at Station ALOHA (Supplementary Figure S7).

At a coarse grained level, the abundance profiles of major marine microbial clades we report here were in general consistent with prior studies at Station ALOHA (DeLong et al., 2006; Bryant et al., 2012, 2016; Mende et al., 2017a) and other comparable oceanic regions (Bork et al., 2015; Lima-Mendez et al., 2015; Sunagawa et al., 2015). The richness and abundance profiles of abundant clades (Figure 1) indicate a positive relationship. This led us to explore the correlations between the two different multi-sample richness indices (core richness and total richness) and total abundance for different phyla (Supplementary Figures S2, S8). We chose to compare different phyla (class for Proteobacteria) rather than clades, as they represent a more consistent taxonomic delineation. Partial correlation analysis showed a strong correlation between phylum abundance and the within-phylum core richness that governed the other relationships (rho = 0.94, partial rho = 0.83, *p* < 10^–20^, Supplementary Table S6). Nevertheless, a significant part of the remaining variation of the phylum abundance could be explained by total within-phylum richness (rho = 0.85, partial rho = 0.55, *p* < 10^–9^). This strong relationship between phyla abundance and diversity of persistent organisms, further reinforces the importance of these core taxa. Although the above-described trend generally held for many different phyla, we observed consistent aberrations from this relationship, in particular Cyanobacteria and Thaumarchaeota had lower within phylum richness than would be expected from their abundances. This was supported by the observation that core-mOTUs assigned to Cyanobacteria (>95% of total Cyanobacteria abundance at all depths above the DCM) and Thaumarchaeota (>95% of total Thaumarchaeota abundance at all depths except at 200 m; 77.5% at 200 m) represented almost the entirety of the total abundance of these clades. In contrast, Alphaproteobacteria showed a much higher within-taxon richness than expected, when considering the total abundance of this group vs. its core-mOTU richness (representing only between 38.2 and 78.6% of the total Alphaproteobacteria abundance at any given depth). This might imply the population structure and diversity in different microbial clades are driven by different evolutionary or environmental forces. Further, our observations in regard to the core and total community as a whole might potentially reflect ongoing sympatric evolution of bacterioplankton in this environment founded upon small number of well-established and persistent populations that provide the foundation for continual diversification of a much greater array of rarer, temporally transient variants. This phenomena may potentially be driven by factors such as more constrained metabolic modalities within Cyanobacteria and Thaumarchaeota, resource partitioning (Hunt et al., 2008), phage predation (Rodriguez-Valera et al., 2009), low intra-population antagonism (Cordero et al., 2012), neutral evolution, as well as other ecological and evolutionary factors operating in tandem (Cordero and Polz, 2014).

**FIGURE 4:**
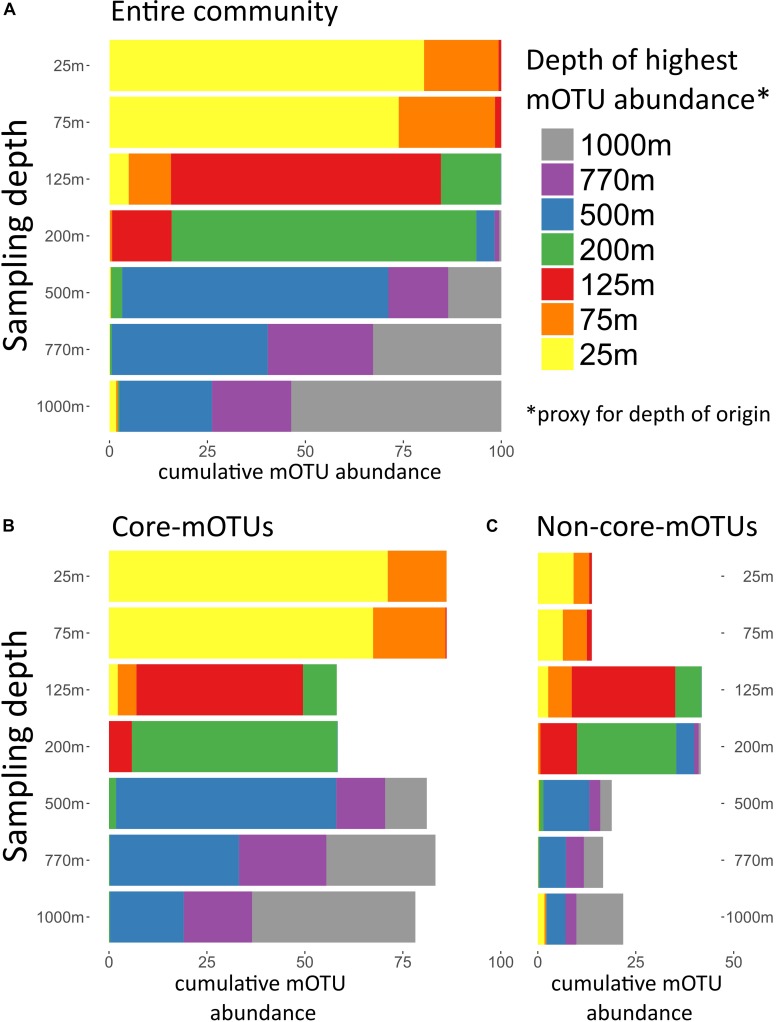
Depth of highest mOTU abundance. Depth of highest mOTU abundance which can be interpreted as a proxy for depth of origin. For every mOTU the depth of highest relative abundance was determined, and mOTUs with the same depth of highest abundance were summed. For each sampled depth, the cumulative abundance of mOTUs assigned to a depth of highest abundance was displayed in different colors. **(A)** Total community, **(B)** only core-mOTUs, and **(C)** only non-core-mOTUs.

The broad trends we observed and their implications for the importance of persistent taxa led us to investigate the depth ranges of different microbial clades and individual mOTUs (Figures 3,4 and Supplementary Figure S4). Only a few microbial clades (SAR11, SAR324 and Euryarchaeota) were represented by at least one core-mOTU at every depth examined, suggesting that cosmopolitan eurybathic clades were in the minority in the water column at Station ALOHA. In support of this, at the fine-grained mOTU level, we did not detect any cosmopolitan taxa (mOTUs) that were present at all depths, confirming that most mOTUs represented depth-stratified phylotypes within their respective clades (Figure 3 and Supplementary Figure S4). Specific mOTUs typically had a single depth stratum in which they were most abundant, with diminishing abundances at bracketing depths (Figure 3). We further investigated the overall depth specificity of mOTUs across the whole microbial community and found that in general, the largest proportion of each depth-specific community was constrained to that given depth (or an adjacent depth stratum with similar environmental properties) (Figure 4A). A focused investigation of the core-mOTUs showed that as a whole, abundant and persistent core phylotypes tended to be even more depth-specific than the total community (Figure 4B). In contrast to a recent report (Mestre et al., 2018), our analyses provided no support for the claim that the most abundant Bacteria and Archaea in the deep ocean were also present in surface waters, or that surface-derived microorganisms thrive at depth. These disparate results might be due to the specific focus on particle attached bacteria of this prior study (Mestre et al., 2018), compared to our focus here on free-living bacterioplankton in the picoplankton size fraction.

Using protein-coding phylogenetic marker genes we surveyed fine-scale diversity patterns in space and time for open ocean bacterioplankton populations. While our analyses confirmed the existence of a high degree of diversity (hundreds of mOTUs within different bacterial lineages), they also suggested that only a small proportion of the total diversity in a given clade was represented by the most abundant and temporally persistent phylotypes. The remainder of the microbial diversity was accounted for by a long “tail” of lower abundance, ephemeral variants, that accounted for most of a given clade’s diversity. Our results indicate that the abundant and persistent core populations form the foundation for diversification of the more ephemeral population variants, that together comprise total clade microdiversity. Due to their overall abundance, we further postulate that the core populations are largely responsible for microbially driven ecosystem processes, and therefore represent ideal targets for more in-depth studies of microbial processes in the open-ocean water column.

## Data Availability Statement

The datasets analyzed for this study can be found in the Supplementary Table S1.

## Author Contributions

DM and ED conceptualized the research and wrote the manuscript. DM and DB performed the analyses.

## Conflict of Interest

The authors declare that the research was conducted in the absence of any commercial or financial relationships that could be construed as a potential conflict of interest.
