# Supplementary material for: Persistent Core Populations Shape the Microbiome Throughout the Water Column in the North Pacific Subtropical Gyre
Source: Front Microbiol. 2019 Oct 1;10:2273. doi: 10.3389/fmicb.2019.02273 (PMC6779783; doi:10.3389/fmicb.2019.02273)
Supplement: Supplementary file 7 [file Data_Sheet_1.pdf]

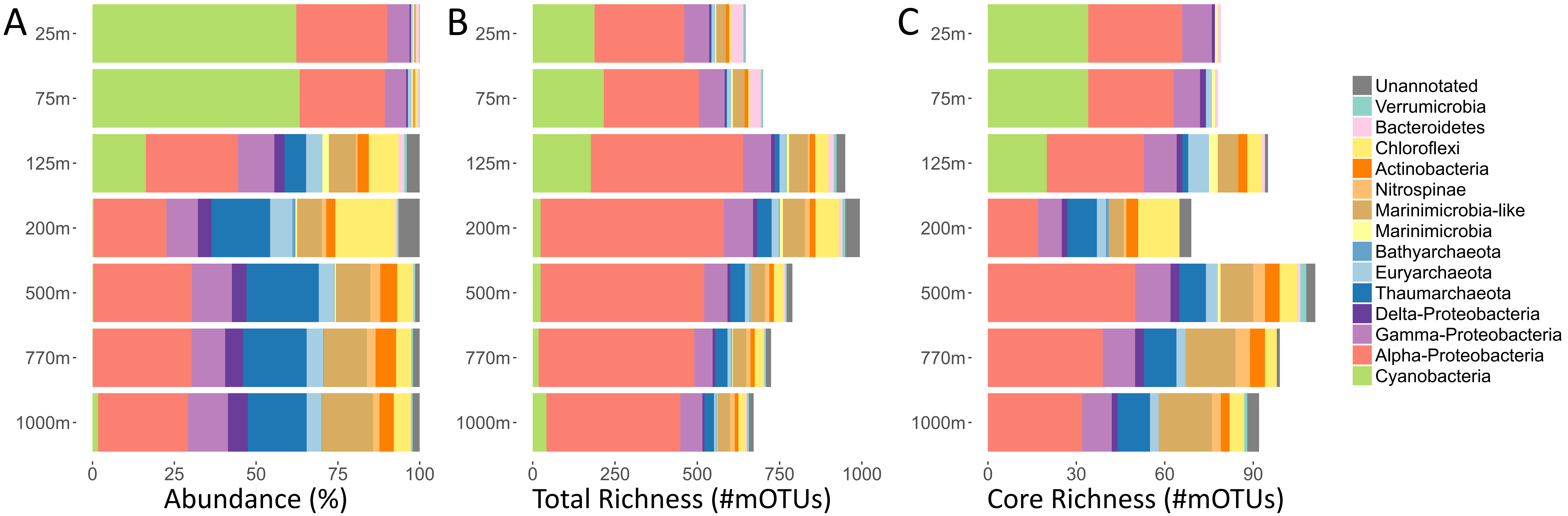

Suppl. Fig. 1: Summary of per depth average abundances and richness indices for different bacterial and archaeal phyla. a) Per depth cumulative abundance in percent of the whole community for each phyla (Abundances are based on COG0012 mOTU abundances and were first summed up on a per sample basis and then averaged across all samples of each depth); b) Per depth total richness (number of COG0012 mOTUs present in at least one sample found at a given depth) plotted by phyla; c) Per depth core richness (number of COG0012 mOTUs present in all samples phyla found at a given depth) plotted by phyla.

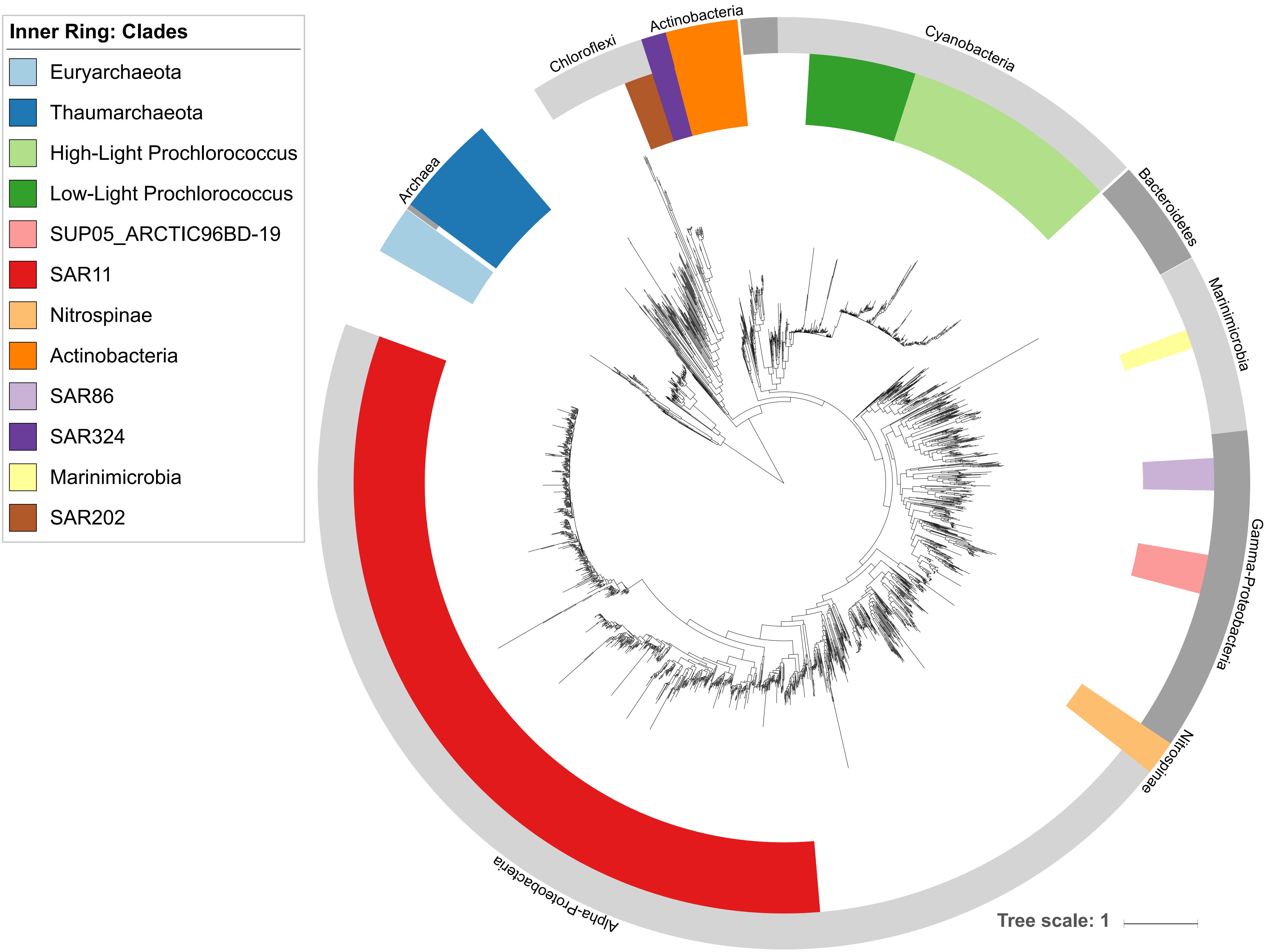

Suppl. Fig. 2: Phylogenetic tree of COG0012 mOTUs found throughout the water column at station ALOHA. The tree was reconstructed from COG0012 sequences extracted from 389 reference genomes and 2228 environmental COG0012 mOTU genes. Bacterial and archaeal groups are labelled according to their taxonomic affiliations (see methods for more details). Clades are annotated using the color scheme explained in the legend in the inner ring. Phyla level annotations are displayed in the outer ring. Clades that represent phylum level clades were colored in both annotation rings. Other phyla were alternatingly colored light and dark grey, and their names are displayed.

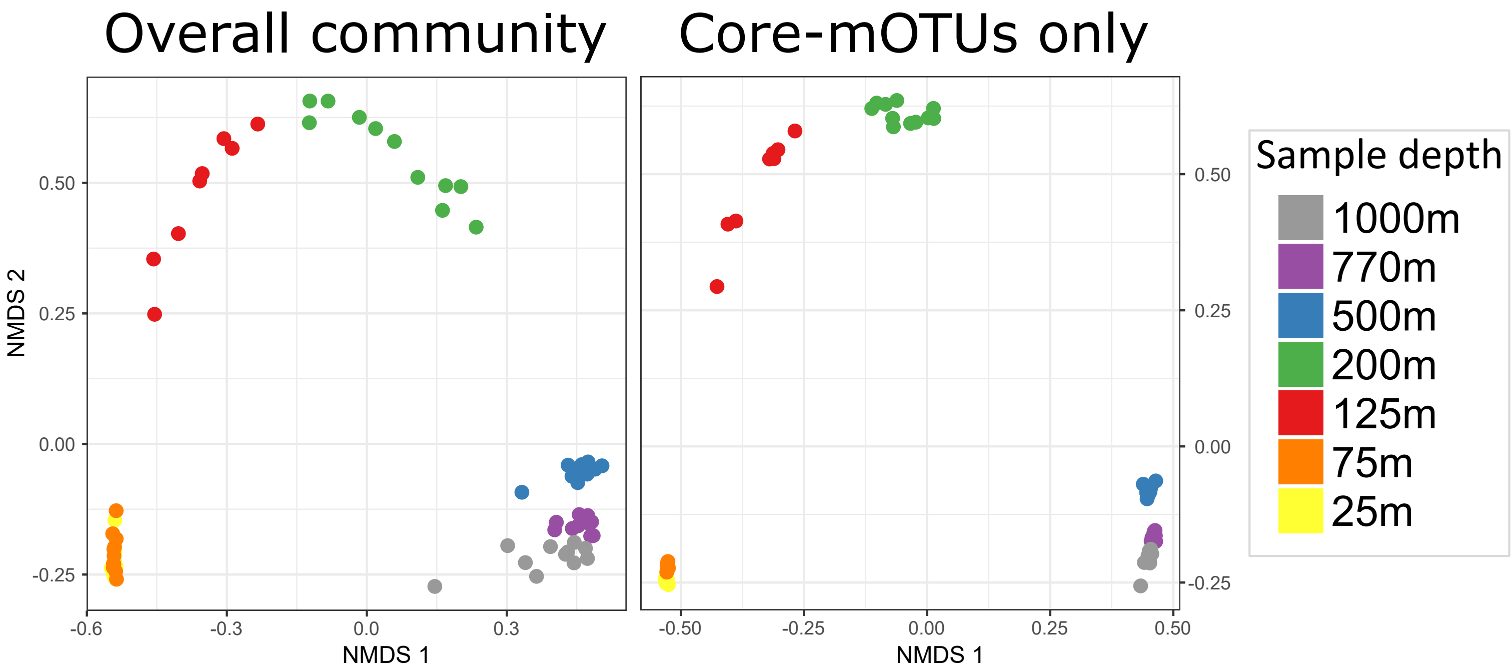

Suppl. Fig. 3. Non-metric multidimensional scaling (NMDS) ordination plot of bacterioplankton COG0012 mOTU abundances across depth and time. Each time point (sample) is displayed by dots colored according to its sampling depth.

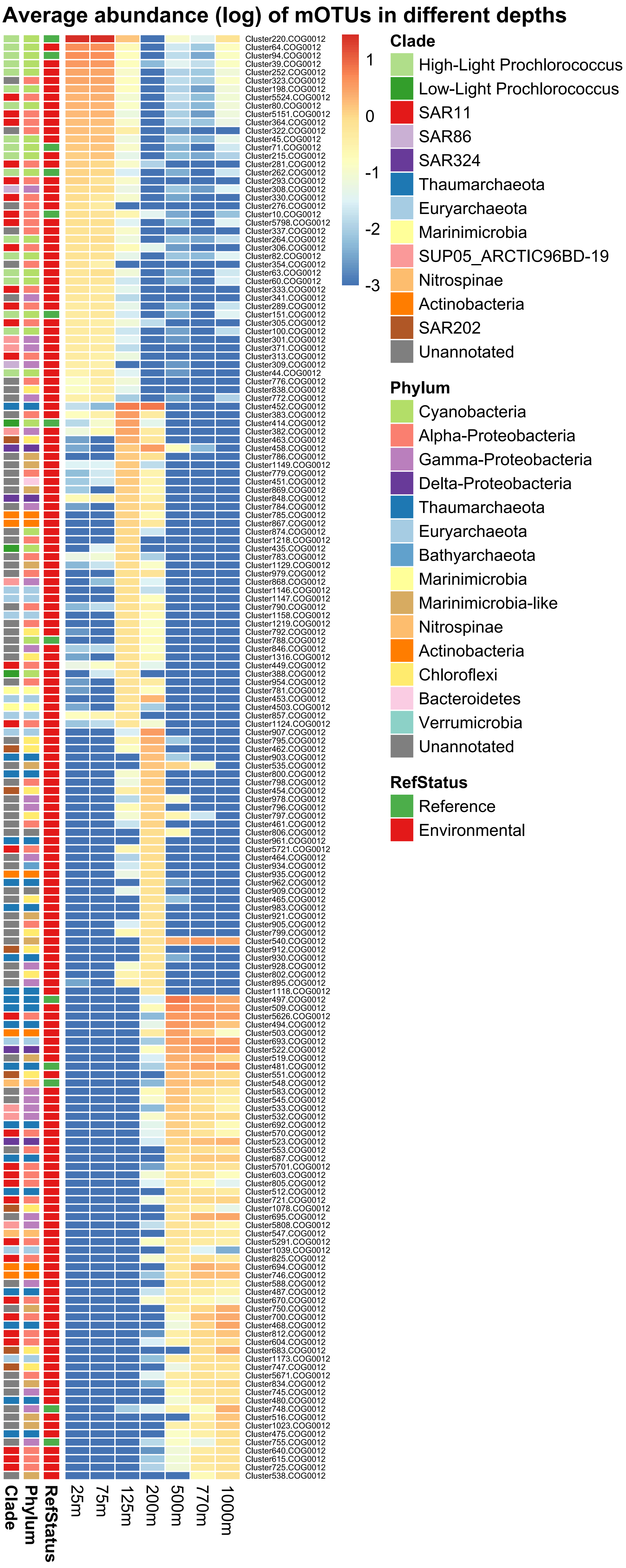

Suppl. Fig. 4: Heatmap of per-depth average core abundances for highly abundant mOTUs. For each mOTU, the average across all samples of each depth was calculated. The 40 most abundant mOTUs for every depth were calculated and plotted in descending order. Side bars (on the left) show if there is a reference genome sequence clustered into the mOTU (RefStatus) and taxonomic annotations to major marine bacterial and archaeal clades. Heatmap colors display the logarithm of the overall abundance in percent of the whole community for each group at each given depth.

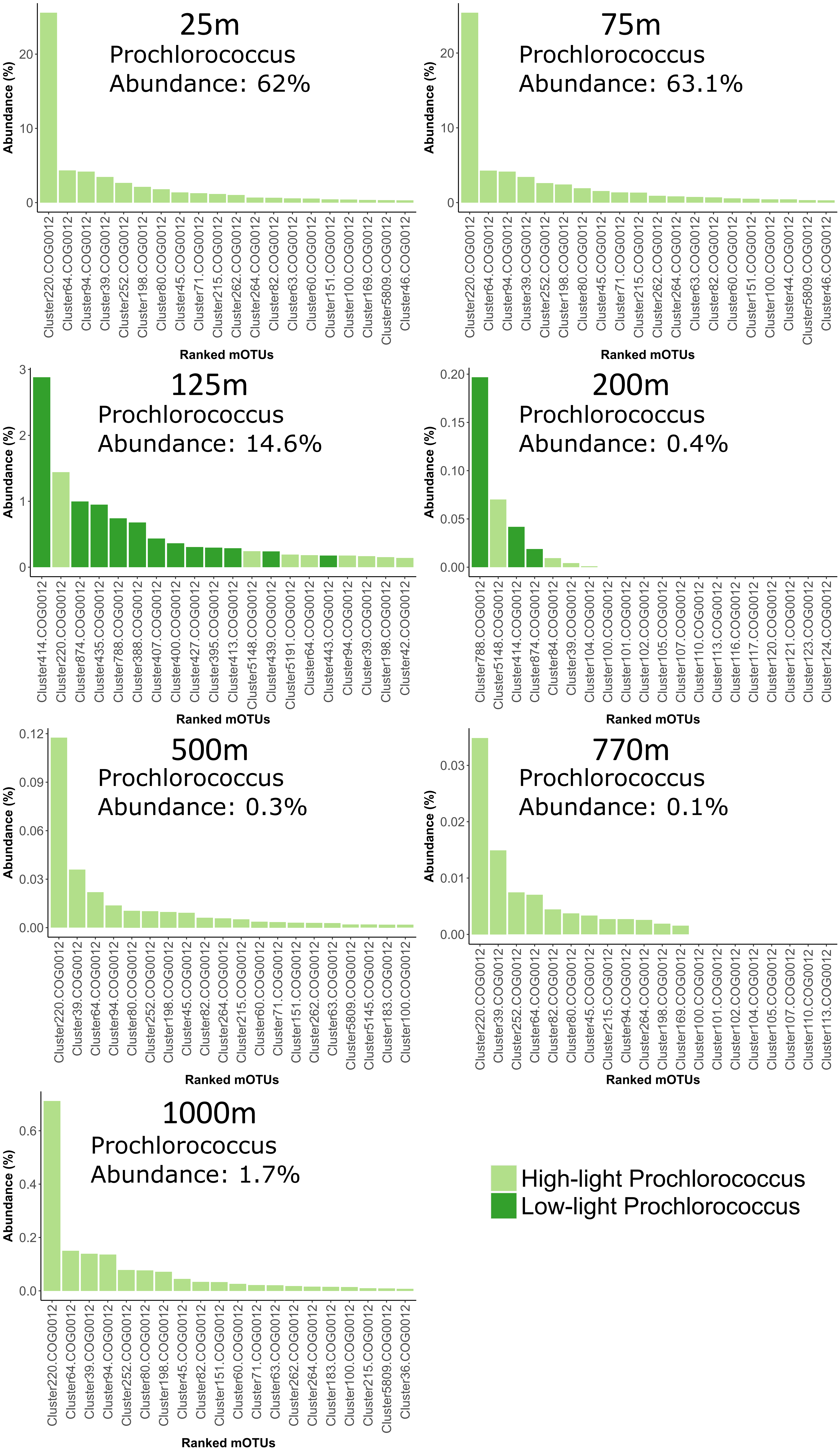

Suppl. Fig. 5: Rank abundance plot of *Prochlorococcus* mOTUs.

mOTU abundances (percent of the whole community) were averaged across all samples of a given depth and the ranked. The 20 most abundant mOTUs are displayed. Bars are colored by *Prochlorococcus*subclade (High-light/Low-light) identity.

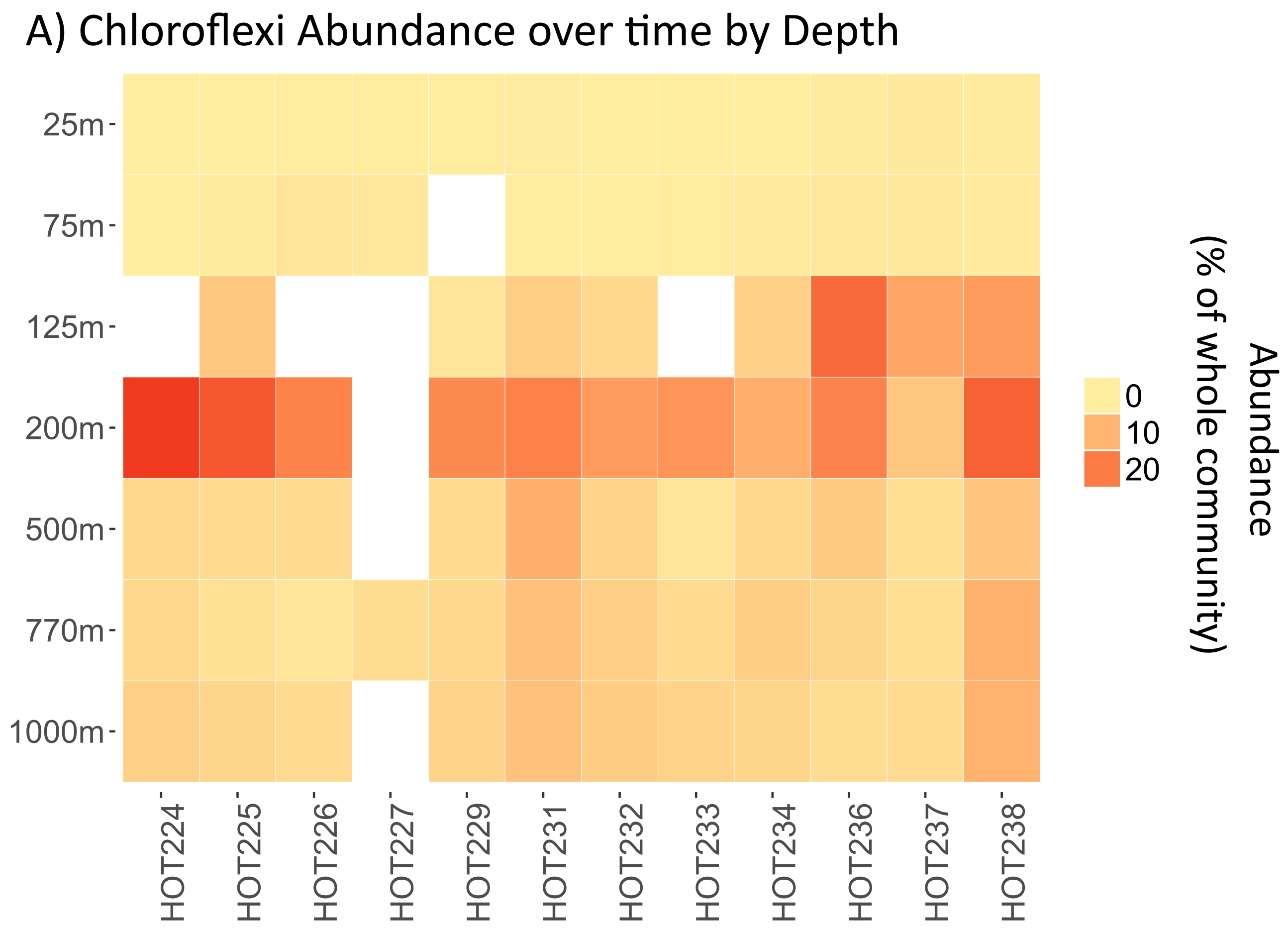

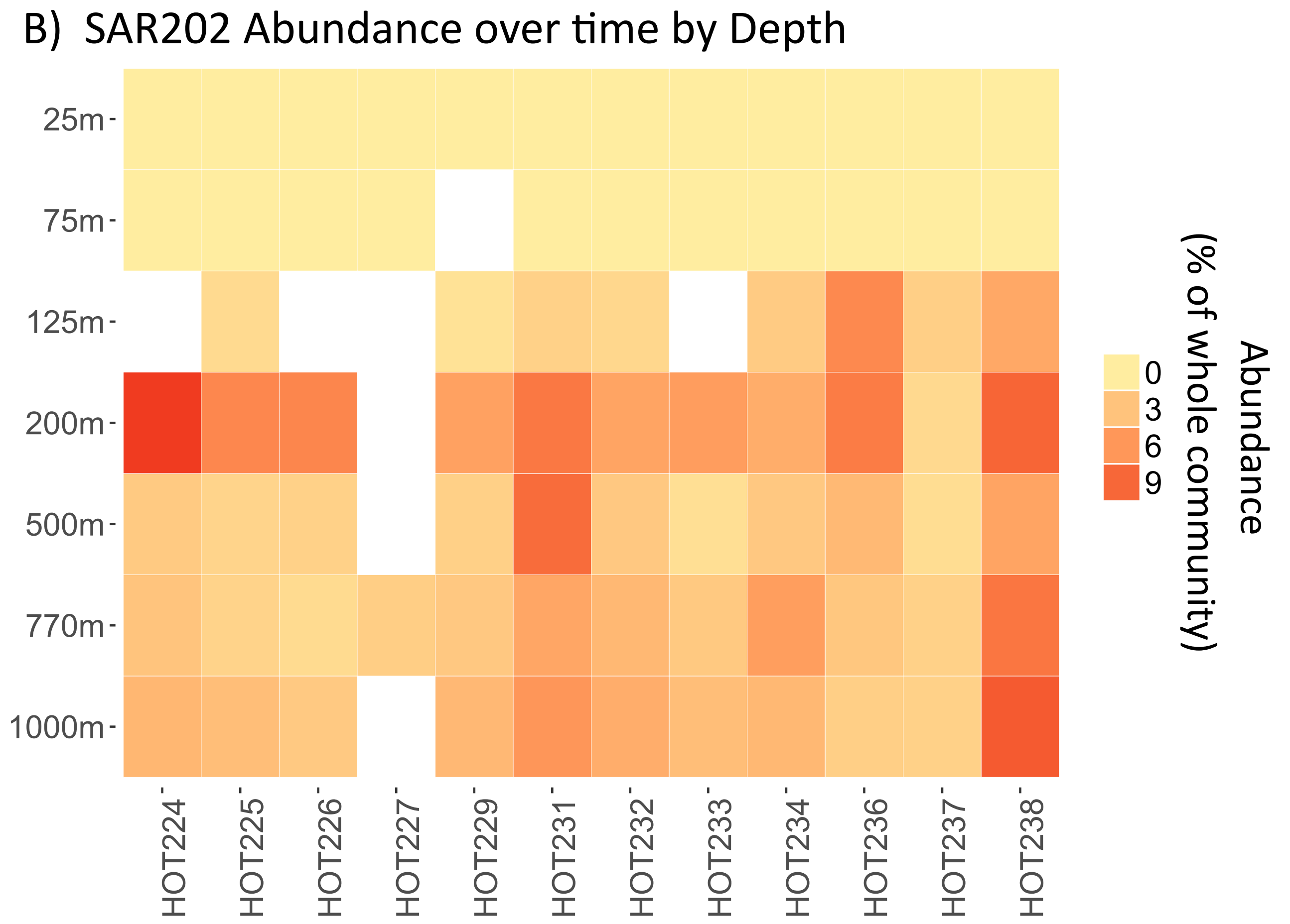

Suppl. Figure 6: Heatmap of Marine Chloroflexi and SAR202 abundances at Station ALOHA by depth
Abundance (reported as percentage of the total microbial community) for A) Marine Chloroflexi and B) SAR202, displayed for each sample by depth and cruise.

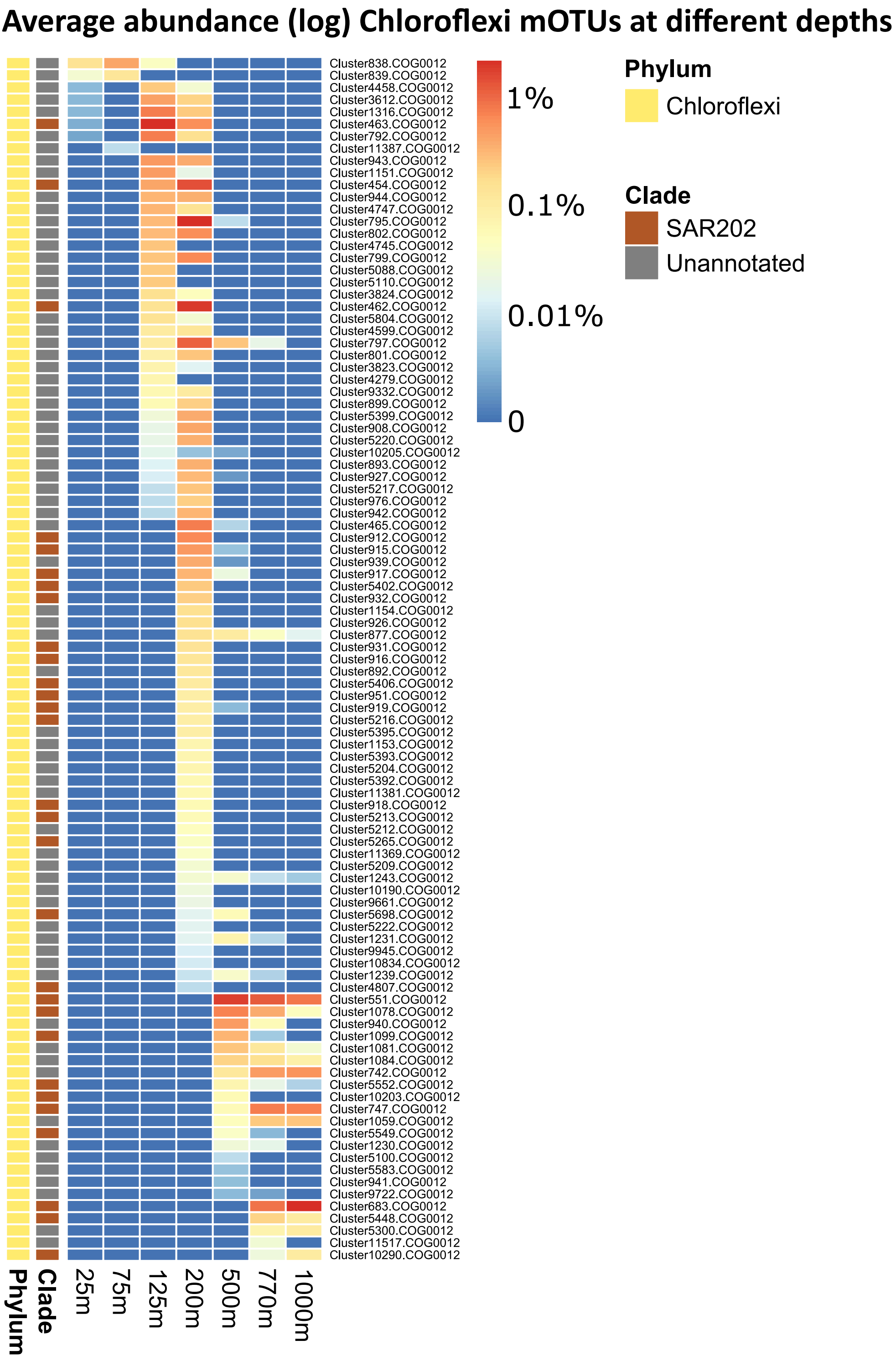

Suppl. Fig. 7: Heatmap of per-depth average core abundances for highly abundant Chloroflexi mOTUs. For each mOTU, the average across all samples of each depth was calculated. The 40 most abundant mOTUs for every depth were calculated and plotted in descending order. Side bars (on the left) taxonomic annotations to major marine bacterial and archaeal clades. Heatmap colors display the logarithm of the overall abundance in percent of the whole community for each group at each given depth.

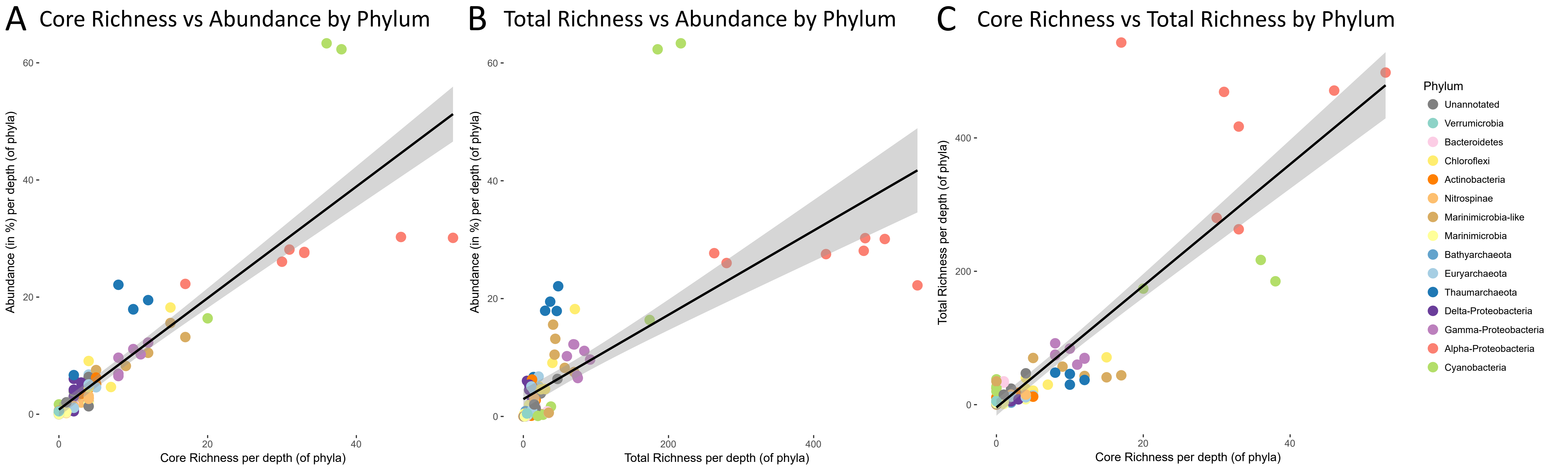

Suppl. Fig. 8: Relationship between core richness, total richness and abundance of major bacterial and archaeal phyla. The total or core richness (inferred from the number of detected mOTUs) within each bacterial and archaeal phylum was calculated for each depth as described in methods, as was the abundance (abundance is reported as percentage of the whole community). Each point represents a summary of the respective data of one phylum across all time-series samples from a given depth. a) Phyla core-richness vs phyla abundance; b) Phyla total richness vs phyla abundance; c) Phyla core richness vs phyla total richness
